# Signaling through Lrg1, Rho1 and Pkc1 Governs *Candida albicans* Morphogenesis in Response to Diverse Cues

**DOI:** 10.1371/journal.pgen.1006405

**Published:** 2016-10-27

**Authors:** Jinglin L. Xie, Nora Grahl, Trevor Sless, Michelle D. Leach, Sang Hu Kim, Deborah A. Hogan, Nicole Robbins, Leah E. Cowen

**Affiliations:** 1 Department of Molecular Genetics, University of Toronto, Toronto, Ontario, Canada; 2 Department of Microbiology and Immunology, Geisel School of Medicine at Dartmouth, Hanover, New Hampshire, United States of America; 3 Aberdeen Fungal Group, School of Medical Sciences, Institute of Medical Sciences, University of Aberdeen, Foresterhill, Aberdeen, United Kingdom; University College Dublin, IRELAND

## Abstract

The capacity to transition between distinct morphological forms is a key virulence trait for diverse fungal pathogens. A poignant example of a leading opportunistic fungal pathogen of humans for which an environmentally responsive developmental program underpins virulence is *Candida albicans*. *C*. *albicans* mutants that are defective in the transition between yeast and filamentous forms typically have reduced virulence. Although many positive regulators of *C*. *albicans* filamentation have been defined, there are fewer negative regulators that have been implicated in repression of filamentation in the absence of inducing cues. To discover novel negative regulators of filamentation, we screened a collection of 1,248 *C*. *albicans* homozygous transposon insertion mutants to identify those that were filamentous in the absence of inducing cues. We identified the Rho1 GAP Lrg1, which represses filamentous growth by stimulating Rho1 GTPase activity and converting Rho1 to its inactive, GDP-bound form. Deletion of *LRG1* or introduction of a *RHO1* mutation that locks Rho1 in constitutively active, GTP-bound state, leads to filamentation in the absence of inducing cues. Deletion of the Rho1 downstream effector *PKC1* results in defective filamentation in response to diverse host-relevant inducing cues, including serum. We further established that Pkc1 is not required to sense filament-inducing cues, but its kinase activity is critical for the initiation of filamentous growth. Our genetic analyses revealed that Pkc1 regulates filamentation independent of the canonical MAP kinase cascade. Further, although Ras1 activation is not impaired in a *pkc1Δ/pkc1Δ* mutant, adenylyl cyclase activity is reduced, consistent with a model in which Pkc1 functions in parallel with Ras1 in regulating Cyr1 activation. Thus, our findings delineate a signaling pathway comprised of Lrg1, Rho1 and Pkc1 with a core role in *C*. *albicans* morphogenesis, and illuminate functional relationships that govern activation of a central transducer of signals that control environmental response and virulence programs.

## Author Summary

The ability to transition between distinct morphologies is a key virulence trait of diverse fungal pathogens. *Candida albicans* is the most common fungal pathogen in humans, causing diseases ranging from superficial skin infections in otherwise healthy adults to invasive, highly morbid infections in immunocompromised hosts. Critical to *C*. *albicans* pathogenesis is its ability to transition between yeast and filamentous forms upon sensing appropriate host-relevant cues. To identify novel negative regulators of morphogenesis, we screened a collection of 1,248 mutants for strains that filament in the absence of inducing cues. We identified Lrg1, a negative regulator of Rho1 activity, as a novel repressor of *C*. *albicans* morphogenesis. Deletion of *LRG1* or introduction of a constitutively active *RHO1* allele promoted polarized cell growth in the absence of inducing cues. Deletion of the Rho1 downstream effector *PKC1* blocked filamentation, in a manner that was independent of the canonical MAP kinase cascade. Instead, we established that Pkc1 functions in concert with the Ras1-cAMP-PKA pathway and enables morphogenesis by stimulating activation the adenylyl cyclase Cyr1, a central signal transducer. Our findings establish a novel signaling pathway critical for the yeast-to-hyphal transition in *C*. *albicans*, and identify novel functional relationships between core pathways that govern morphogenesis.

## Introduction

Fungi are ubiquitous in the environment and are among the most pervasive opportunistic pathogens. *Candida albicans*, which is a common constituent of the human mucosal microbiota, is found asymptomatically in the oral cavity, gastrointestinal tract and genital area of many individuals [1, 2]. However, *C*. *albicans* is among the leading pathogens of humans where in patients with compromised immunity, it can cause superficial oral and vaginal infections. If it enters the bloodstream or reaches vital organs, the infection can be fatal with mortality rates approaching 40% [3]. Fundamental to *C*. *albicans* pathogenesis is its ability to transition between yeast and filamentous forms, including hyphae and pseudohyphae. During the early stages of infection, *C*. *albicans* is disseminated in the yeast form, and filamentation is important for tissue penetration and escape from host immune cells [4]. Mutants unable to undergo morphological transitions typically have reduced virulence [5–7], and pharmacological inhibitors of filamentation show efficacy *in vivo* as potential therapeutics [8]. Thus, the genetic circuitry controlling morphogenetic transitions provides a rich source of virulence factors that expands the target space for antifungal drug development and the potential chemical diversity of therapeutics, as these are typically overlooked by conventional screening strategies.

The transition from yeast to filamentous growth in *C*. *albicans* is triggered by diverse host relevant cues, including serum, nutrient limitation, alkaline pH, and elevated temperature [9, 10]. This process is governed by a complex network of signaling pathways, many of which are involved in activating the filamentous growth program [9, 10]. For example, the small GTPase Ras1 acts with adenylyl cyclase (Cyr1) to activate the cAMP-PKA pathway [11], a signaling cascade of central importance for *C*. *albicans* morphogenesis, that culminates in the activation of the terminal transcription factor Efg1 [9, 12]. Furthermore, Ras1 signals through mitogen-activated protein (MAP) kinases upstream of the transcription factor Cph1, including Ste11, Hst7, and Cek1/Cek2, to coordinate filamentous growth specifically in response to nutrient deprivation [13, 14]. Many of the filament-inducing cues also require a concomitant increase in temperature to relieve repression of morphogenetic signaling mediated by the molecular chaperone Hsp90 [15]. Additionally, morphogenesis in *C*. *albicans* is negatively regulated by a number of transcription factors, including Sfl1, Tup1, Nrg1, and Rfg1 [9]. Recent genome-scale analyses of morphogenesis have identified many novel regulators of filamentous growth in both *C*. *albicans* and the model yeast *Saccharomyces cerevisiae* [16, 17], primarily focusing on positive regulators of filamentation. Hence, the vast complexity of the regulatory network governing morphogenesis is only beginning to be appreciated.

Cellular morphogenesis in *C*. *albicans* is also contingent upon extensive remodeling of the fungal cell wall. This highly dynamic process involves loosening of the cell wall by digestive enzymes such as glucanases and chitinases, and directing cell wall synthesis towards a specific region on the cell surface [18]. It is well established that Rho1 is a master regulator of the cell wall integrity signaling cascade [19]. It is the regulatory subunit of β-1,3-D-glucan synthase, and directly controls cell wall biosynthesis via the binding and activation of the catalytic subunits, Fks1 and Fks2 [20]. Rho1 is also responsible for orchestrating changes in the cell wall in response to various forms of cell wall stress by activating the Pkc1-dependent MAP kinase cascade that includes Bck1, Mkk2 and Mkc1 [18]. Like other small GTPases, Rho1 cycles between an active GTP-bound state and an inactive GDP-bound state. It is activated by guanine nucleotide exchange factors (GEFs), such as Rom2, and inactivated by GTPase-activating proteins (GAPs), such as Lrg1 [21]. In its active form, Rho1 signals through interaction with downstream effectors such as Pkc1 [21]. Despite the critical role of Rho1 in cell wall biogenesis, none of its regulators or effectors have been implicated in *C*. *albicans* morphogenesis.

In order to expand our understanding of the complex *C*. *albicans* morphogenetic network, we screened a collection of 1,248 *C*. *albicans* homozygous transposon insertion mutants to identify negative regulators of filamentation. We identified the Rho1 GAP Lrg1 as a novel repressor of the filamentous growth program. Deletion of *LRG1* or introduction of a *RHO1* mutation that locks Rho1 in a constitutively active GTP-bound state, led to filamentation in the absence of inducing cues. Deletion of the Rho1 downstream effector *PKC1* completely blocked filamentous growth in response to numerous cues, implicating this kinase as a key regulator of *C*. *albicans* filamentation. We found that the kinase activity of Pkc1 was critical for the initiation of filamentous growth. Through genetic analyses, we discovered that Pkc1 regulates filamentation independent of its canonical MAP kinase cascade. We further established that loss of Pkc1 does not impair activation of Ras1 in response to filament-inducing cues, but does reduce activation of the adenylyl cyclase. Our results support a model in which Pkc1 acts as a global regulator of *C*. *albicans* morphogenesis in part through the regulation of adenylyl cyclase and in part through a cAMP-independent pathway. Thus, we establish a new role for Lrg1, Rho1, and Pkc1 in controlling *C*. *albicans* morphogenesis in response to diverse cues, and unveil a novel functional relationship between two central signal transduction cascades that govern cellular morphogenesis.

## Results

### A genomic screen for morphogenetic regulators identifies Lrg1 as a novel repressor of filamentation

**Fig 1:**
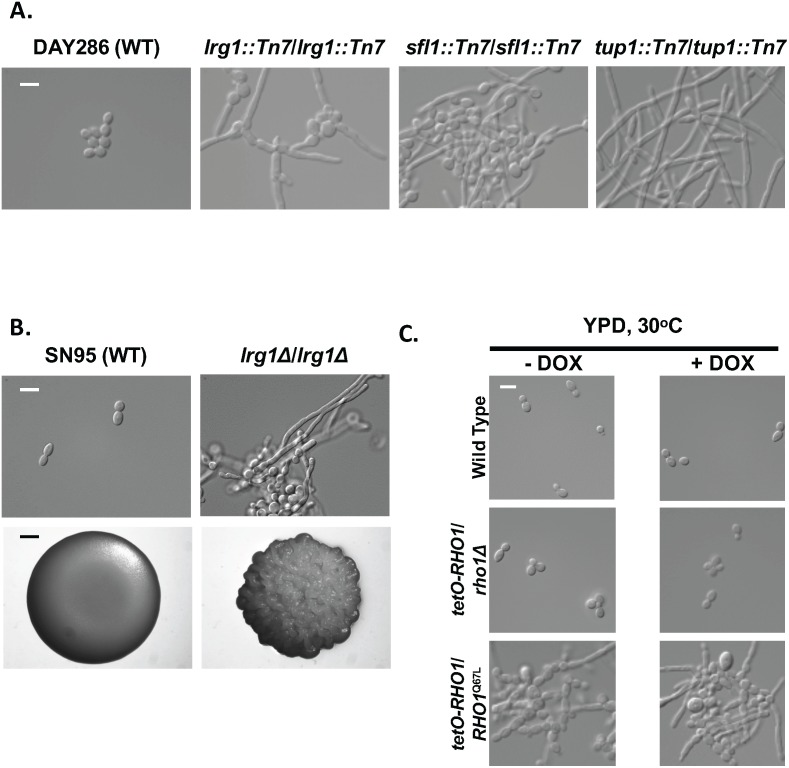
Lrg1 negatively regulates filamentation via Rho1. **(A)** Lrg1 is a repressor of filamentous growth. Strains were subcultured to log phase in YPD at 30°C for 4 hrs, and cells were imaged by DIC microscopy. The scale bar indicates 10 μm. **(B) Top panel:** Strains were subcultured to log phase in YPD at 30°C for 4 hrs, and cells were imaged by DIC microscopy. The scale bar indicates 10 μm. **Bottom panel:** 1 μL of a saturated overnight culture was spotted on YPD plates. Plates were incubated at 30°C for 48 hrs and images were taken using a Zeiss stereoscope. Scale bar indicates 1 mm. **(C)** Activated Rho1 promotes filamentation. Overnight cultures were subcultured for 24 hrs in the presence or absence of 0.05 μg/ml of doxycycline to achieve transcriptional repression of the wild-type allele of *RHO1* that is under the control of the *tetO* promoter. Strains were subcultured in YPD at 30°C, with or without 0.05 μg/ml of doxycycline, for 4 hrs. Cells were imaged by DIC microscopy. The scale bar indicates 10 μm.

Given that a key attribute of *C*. *albicans* pathogenesis is its ability to transition between yeast and filamentous forms and that there is a limited appreciation of negative regulators of filamentation beyond cell cycle regulators and transcriptional repressors, we screened a collection of homozygous transposon insertion mutants to identify genes that encode repressors of morphogenesis [22, 23]. We scored mutants based on their degree of filamentous growth in the absence of filament-inducing cues (S1 Table), and upon validation, identified three negative regulators of filamentation: Tup1, Sfl1, and Lrg1 (Fig 1A). Both Tup1 and Sfl1 have been characterized extensively as transcriptional repressors of hyphal growth [24, 25], validating our screen. In contrast, Lrg1 is largely uncharacterized in *C*. *albicans* and to our knowledge has not been implicated in morphogenesis. To verify the phenotype of the *lrg1*::*Tn7*/*lrg1*::*Tn7* transposon mutant, we engineered a homozygous *LRG1* deletion mutant, and found that this strain was also hyper-filamentous in the absence of any inducing cue (Fig 1B). Hence, Lrg1 acts as a novel repressor of filamentous growth in *C*. *albicans*.

### Lrg1 represses *C*. *albicans* filamentation via Rho1

In *S*. *cerevisiae* the Lrg1 ortholog is a Rho1 GAP that serves as a negative regulator of cell wall integrity [26, 27], cell separation [28], and pseudohyphal growth [28]. Given that very little is known about *RHO1* regulating *C. albicans* morphogenesis, we wanted to determine if the effect of *LRG1* on filamentous growth was mediated by Rho1 signaling. We generated a strain in which we deleted one allele of *RHO1* and placed the only remaining allele under the control of the doxycycline-repressible *tetO* promoter. In the absence of doxycycline, *RHO1* was overexpressed in the *tetO-RHO1/rho1*Δ strain (S1 Fig), but this did not alter cellular morphology compared to wild type (Fig 1C). Doxycycline-mediated transcriptional repression of *RHO1* with 0.05 μg/ml also had no impact on morphogenesis (Fig 1C and S1 Fig). This low concentration of doxycycline has been optimized to enable transcriptional repression of essential genes under the control of the *tetO* repressible promoter system while minimizing effects on cellular viability [29]. Since Rho1 is known to cycle between an active GTP-bound state and an inactive GDP-bound state, we replaced the deleted *RHO1* allele in the *tetO-RHO1*/*rho1Δ* strain with *RHO1*^*Q67L*^ in order to lock Rho1 in its active conformation [30]. Introduction of the *RHO1*^*Q67L*^ allele induced filamentation in the absence of inducing cues (Fig 1C), demonstrating that activated Rho1 promotes polarized growth. Thus, our results support a model in which Lrg1 acts as a Rho1 GAP in *C*. *albicans*, driving Rho1 into its GDP-bound form and repressing the yeast-to-filament transition.

### Pkc1 is a master regulator of morphogenesis

**Fig 2:**
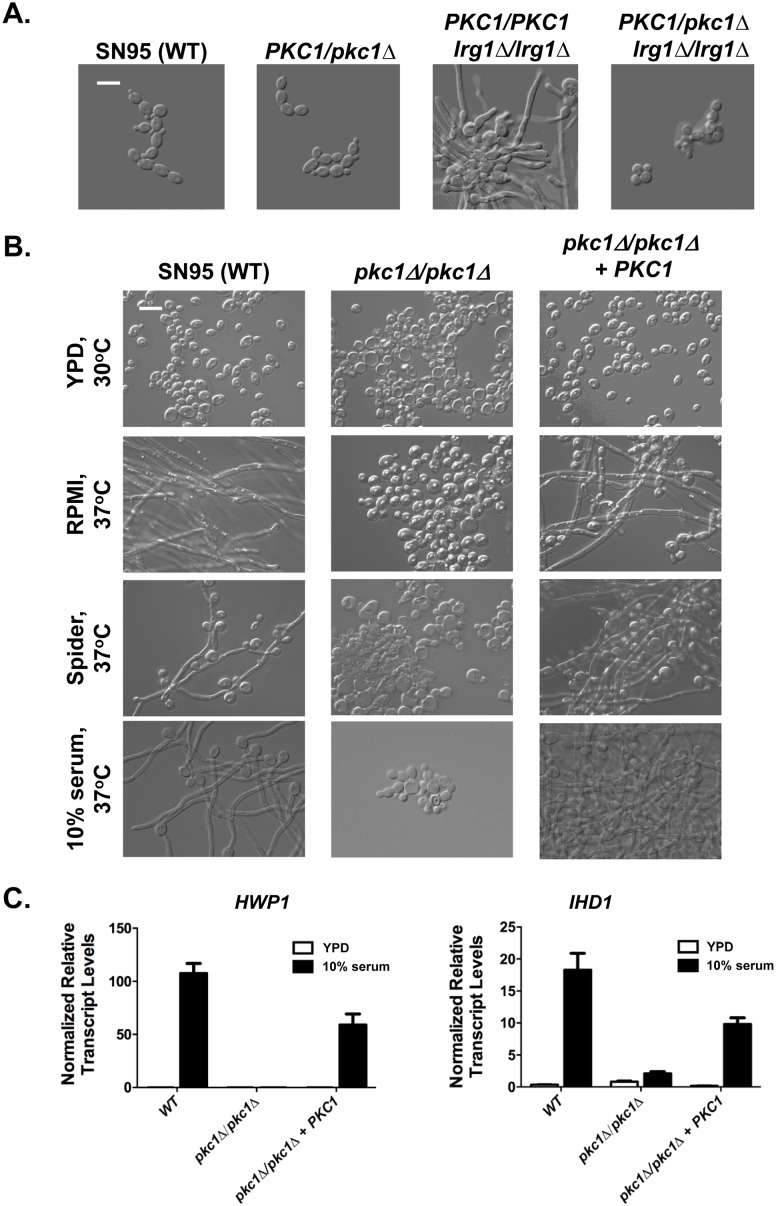
Pkc1 is a master regulator of filamentous growth. **(A)** Pkc1 acts downstream of Lrg1. Strains were subcultured to log phase in YPD at 30°C for 4 hrs. Cells were imaged by DIC microscopy. The scale bar indicates 10 μm. **(B)** Homozygous deletion of *PKC1* blocks filamentation in response to diverse cues. Strains were subcultured to log phase in the specified conditions for 4 hrs. Cells were imaged by DIC microscopy. The scale bar indicates 10 μm. **(C)** Deletion of *PKC1* blocks the upregulation of filament-specific transcripts *HWP1* and *IHD1*. Strains were subcultured to log phase in YPD at 30°C or YPD + 10% serum at 37°C, and the transcript levels of *HWP1* and *IHD1* was monitored by qRT-PCR and normalized to *GPD1*. Data are plotted as means ± SD for triplicate samples and are representative of two independent experiments.

Next, we set out to dissect the mechanism through which Rho1 promotes filamentation. The most well characterized effector downstream of Rho1 is the protein kinase Pkc1, a central protein kinase that has not been implicated in *C*. *albicans* morphogenesis. To determine if Rho1 signals through Pkc1 to mediate filamentous growth, we deleted one allele of *PKC1* in both a wild-type strain and an *lrg1*Δ*/lrg1*Δ mutant. We predicted that deletion of *PKC1* would block the hyperfilamentous phenotype of the *lrg1*Δ*/lrg1*Δ mutant. Deletion of one allele of *PKC1* had no impact on morphogenesis in the wild-type strain, but completely blocked filamentation induced by *LRG1* deletion in the absence of filament-inducing cues (Fig 2A). This epistatic relationship positions Pkc1 downstream of Lrg1 and Rho1 in governing the yeast-to-hyphal transition.

To further explore the role of Pkc1 in morphogenesis, we characterized the ability of a homozygous *PKC1* deletion mutant to undergo filamentous growth. We found that the *pkc1*Δ*/pkc1*Δ mutant was unable to filament in response to various filament-inducing cues, including RPMI medium, carbon-limiting Spider medium, and serum (Fig 2B). Filamentation was rescued upon complementation with a wild-type allele of *PKC1* (Fig 2B). Further, we performed qRT-PCR to monitor the expression of hyphal-specific genes *HWP1* and *IHD1* [31] in the wild-type strain, *pkc1*Δ*/pkc1*Δ mutant, and complemented *PKC1* strain grown in serum at 37°C. Consistent with the morphology observed by microscopy, deletion of *PKC1* blocked the upregulation of *HWP1* and *IHD1*, and complementation of *PKC1* partially rescued induction of these transcripts (Fig 2C). Since strains lacking Pkc1 have been reported to have cell wall defects that require the addition of sorbitol for osmotic support [32], we tested whether filamentation of a *pkc1*Δ*/pkc1*Δ mutant could be restored in the presence of sorbitol. Strikingly, we found that the addition of sorbitol as an osmotic stabilizer did not rescue filamentation of the mutant lacking *PKC1*, suggesting that its inability to filament cannot be attributed solely to a severe cell wall defect (S2 Fig). Together, our results suggest that Rho1-mediated activation of Pkc1 is required for morphogenesis in response to diverse cues, and thus we identify a novel central regulator of filamentation in *C*. *albicans*.

### Pkc1 kinase activity is important for the initiation of filamentation

**Fig 3:**
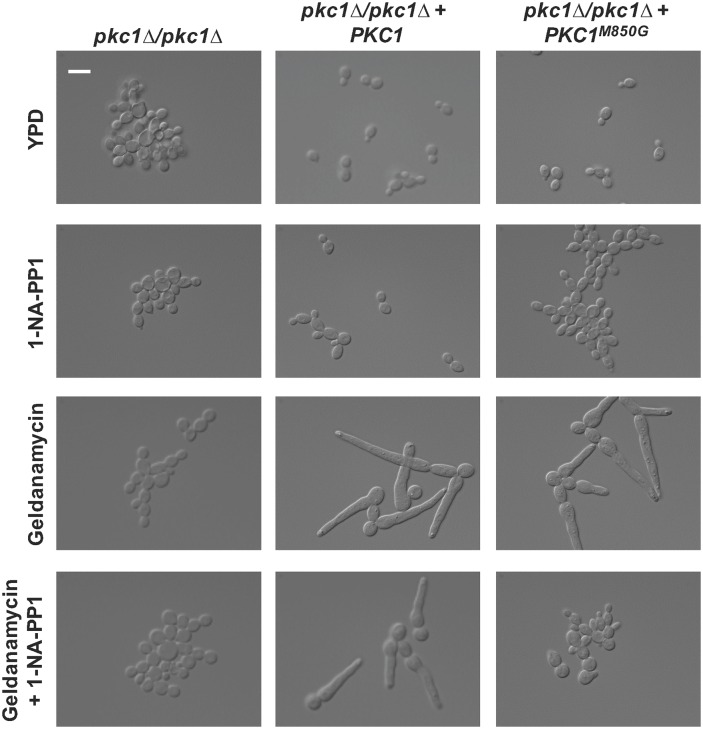
Pkc1 kinase activity is critical for filamentation. Strains were subcultured for 4 hrs with either 10 μM of the Hsp90 inhibitor geldanamycin, 5 μM of the ATP analog 1-NA-PP1 that inhibits the gatekeeper allele, or both. Cells were imaged by DIC microscopy. The scale bar indicates 10 μm.

To elucidate the specific role of Pkc1 in morphogenesis, we tested whether Pkc1 kinase activity is required for the initiation of filamentation. To do so, we constructed a strain in which the *pkc1Δ/pkc1Δ* mutant is complemented with *PKC1*^*M850G*^, a gatekeeper allele that renders the kinase susceptible to inhibition by the ATP analog 1-Naphthyl-PP1 (1-NA-PP1) without affecting its kinase activity in the absence of the inhibitor [33]. We validated that this allele functions as expected, as 1-NA-PP1 treatment phenocopied deletion of *PKC1* in conferring hypersensitivity to calcofluor white in a strain with the gatekeeper allele as its only source of *PKC1* (*pkc1Δ/pkc1Δ* + *PKC1*^*M850G*^) (S3 Fig). This pharmacological inhibitor had no impact on a strain harboring wild-type *PKC1* (S3 Fig). Thus, we were able to specifically interrogate the impact of Pkc1 kinase activity on filamentation. Since the Pkc1 gatekeeper strain is sensitive to elevated temperatures (S3 Fig), we utilized the Hsp90 inhibitor geldanamycin as a cue that induces filamentation at 30°C [15]. In the absence of 1-NA-PP1, treatment with geldanamycin alone induced filamentation in the *pkc1Δ/pkc1Δ* mutant complemented with either a wild-type *PKC1* allele or the *PKC1*^*M850G*^ allele (Fig 3). Treatment with 1-NA-PP1 had no impact on strains lacking the gatekeeper allele, but completely blocked filamentation induced by geldanamycin in the *pkc1Δ/pkc1Δ* mutant complemented with the *PKC1*^*M850G*^ allele (Fig 3). Our results demonstrate that Pkc1 kinase activity is critical for the establishment of filamentous growth.

### Pkc1 activates cAMP signaling to enable filamentation

**Fig 4:**
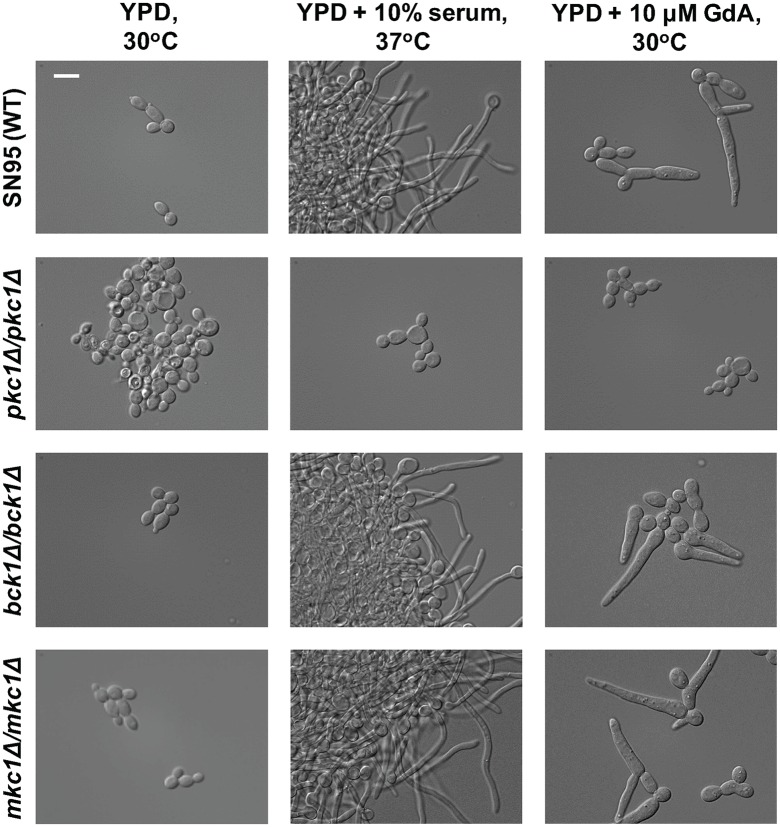
The MAP kinase cascade downstream of Pkc1 is not required for filamentous growth. Strains were subcultured for 4 hrs in YPD at 30°C, YPD + 10% serum at 37°C, or YPD + 10μM geldanamycin (GdA: Hsp90 inhibitor) at 30°C. Cells were imaged by DIC microscopy. The scale bar indicates 10 μm.

Pkc1 signaling regulates multiple targets in *C*. *albicans*, most notably the mitogen-activated protein kinase (MAPK) cascade, which is comprised of a linear series of protein kinases including Bck1, Mkk2, and Mkc1 [34]. To determine if Pkc1 enables filamentous growth through this signaling cascade, we generated homozygous deletion mutants and monitored their ability to filament in response to multiple inducing cues. Unlike the *pkc1*Δ*/pkc1*Δ mutant, homozygous deletion mutants lacking *BCK1* or *MKC1* were not defective in filamentation in response to the Hsp90 inhibitor geldanamycin at 30°C or in response to serum at 37°C (Fig 4), suggesting that Pkc1 regulates *C*. *albicans* polarized growth through targets distinct from the MAPK cascade.

**Fig 5:**
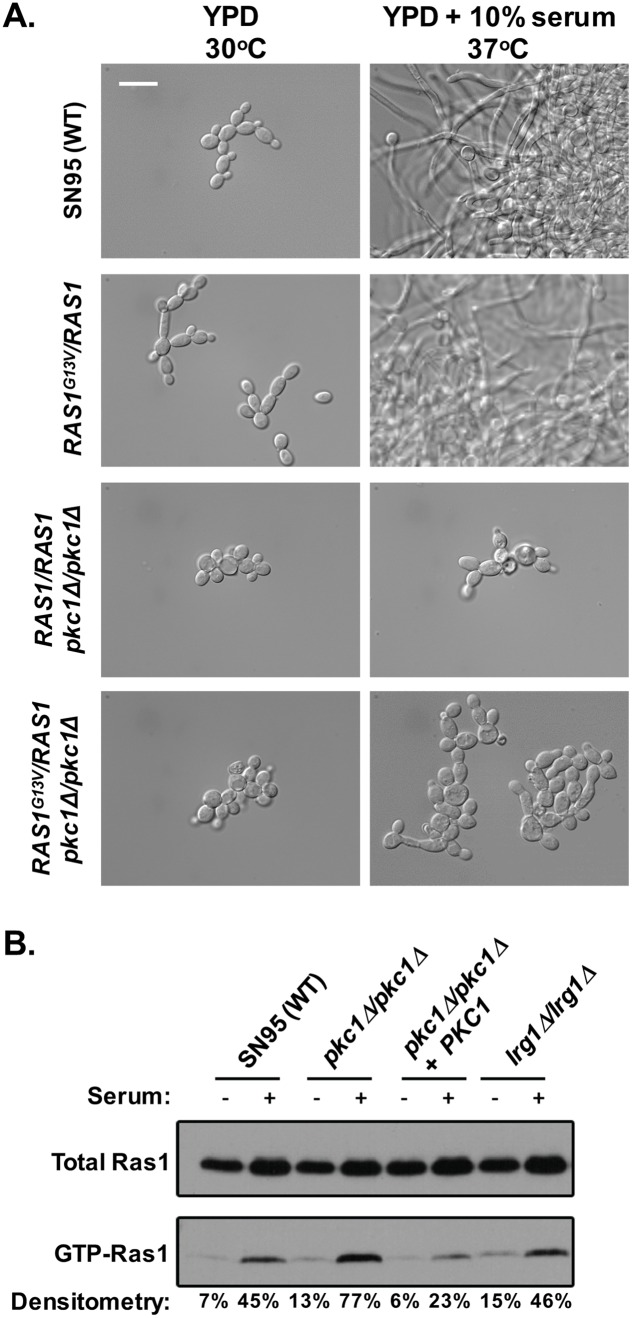
Pkc1 acts downstream of Ras1 to regulate filamentation. **(A)** Hyperactivation of Ras1 does not rescue filamentation in a mutant lacking Pkc1. Strains were grown in YPD at 30°C or YPD + 10% serum at 37°C for 3.5 hrs. Cells were imaged by DIC microscopy. The scale bar indicates 15 μm. **(B)** Deletion of *PKC1* does not affect Ras1 activation. Strains were grown in YPD at 30°C or YPD + 10% serum at 37°C for 3.5 hrs. The total Ras1 protein and GTP-Ras1 fraction were resolved by SDS-PAGE gel. The GTP-Ras1:total Ras1 ratio is shown as a percentage.

The role of Pkc1 as a positive regulator of morphogenesis in response to diverse cues is reminiscent of components of the cAMP-PKA signaling cascade that are activated in part by the GTPase Ras1 [15, 35]. To test the hypothesis that Pkc1 promotes filamentous growth through regulation of cAMP-PKA signaling, we performed a genetic epistasis experiment by introducing a dominant active *RAS1*^*G13V*^ allele into the *pkc1Δ/pkc1Δ* mutant. The dominant *RAS1*^*G13V*^ mutation enhances filamentation under permissive conditions without affecting morphology in rich medium at 30°C [12, 35]. If Pkc1 functions downstream of Ras1 to execute polarized growth programs, then the *RAS1*^*G13V*^ allele should not be able to rescue filamentation in the *pkc1Δ/pkc1Δ* mutant, and activation of wild-type Ras1 should occur independently of Pkc1. Alternatively, if Pkc1 acts upstream of Ras1, then the *RAS1*^*G13V*^ allele should rescue filamentation in the *pkc1Δ/pkc1Δ* mutant, and activation of wild-type Ras1 would require Pkc1 activity. We found that hyperactivation of the Ras1 pathway via the *RAS1*^*G13V*^ mutation was not sufficient to restore filamentation in the absence of *PKC1* (Fig 5A). Further, Ras1 activation in response to serum at 37°C was unaffected by deletion of *PKC1*, as measured by levels of GTP-Ras1 (Fig 5B), suggesting that Pkc1 does not function upstream of Ras1. Notably, deletion of *LRG1* did not lead to hyperactivation of Ras1 despite causing constitutive filamentous growth (Fig 5B), further supporting a model that Lrg1 negatively regulates filamentation specifically through Pkc1.

**Fig 6:**
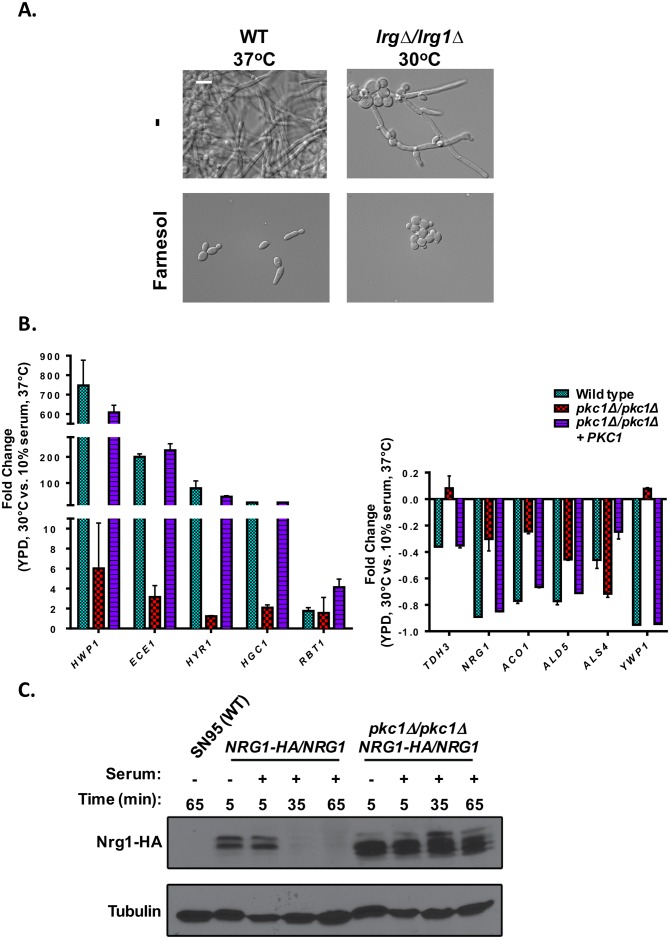
Pkc1 acts upstream of cAMP signaling. **(A)** Farnesol inhibits filamentation induced by *LRG1* deletion. Strains were grown in YPD at 30°C or YPD at 37°C, in the absence or presence of 200 μM farnesol for 3.5 hrs. Cells were imaged by DIC microscopy. The scale bar indicates 10 μm. **(B)** Cyr1-dependent changes in gene expression require functional Pkc1. Strains were grown in YPD at 30°C or YPD + 10% serum at 37°C for 3.5 hrs at 200 rpm. Total RNA was analyzed on the nanostring ncounter system. Shown is the fold change of expression at 30°C vs. 37°C. Data are plotted as means ± SD for two independent experiments. (C) Cyr1-dependent Nrg1 degradation requires functional Pkc1. Strains were grown in YPD at 30°C or YPD + 10% serum at 37°C for 5 min, 35 min, or 65 min. Total proteins were resolved by SDS-PAGE gel and the blot was hybridized with α-HA to detect Nrg1 and α-tubulin to monitor tubulin as loading control.

Active Ras1 participates in the stimulation of the adenylyl cyclase activity of Cyr1 to produce cAMP, which activates the catalytic subunits of PKA [36]. To further probe if the Lrg1-Rho1-Pkc1 cascade acts downstream of Ras1 in the cAMP-PKA cascade to regulate filamentous growth, we exploited the quorum-sensing molecule farnesol. Farnesol blocks filamentation through the inhibition of Cyr1 [37]. If Pkc1 acts at or above Cyr1, then deletion of *LRG1* should not be able to induce filamentous growth in the presence of farnesol. In contrast, if Pkc1 acts below Cyr1 or independently of the Ras1-PKA regulators, then we would expect a homozygous *LRG1* mutant to maintain its filamentous growth phenotype in the presence of farnesol. We observed that inhibition of cAMP signaling with farnesol blocked filamentation caused by homozygous deletion of *LRG1*, suggesting that Pkc1 acts below Ras1 but above or at Cyr1 to regulate *C*. *albicans* morphogenesis (Fig 6A). To determine if Cyr1 can be activated in response to filament-inducing cues in the absence of Pkc1, we used Nanostring technology to monitor the expression of Cyr1 reporter genes in response to serum in a wild-type strain and a *pkc1Δ/pkc1Δ* mutant [11]. Strikingly, homozygous deletion of *PKC1* blocked the upregulation of filament-specific transcripts, such as *HWP1* and *HGC1*, and the downregulation of yeast-specific transcripts, such as *YWP1* and *NRG1* (Fig 6B and S4 Fig), suggesting that Cyr1 activity and cAMP signaling are impaired in the absence of Pkc1. Since cAMP signaling is also required for Nrg1 protein degradation in response to filament-inducing cues [38], we compared the protein levels of Nrg1 in a wild-type strain and *pkc1Δ/pkc1Δ* mutant in response to serum. In a wild-type strain, levels of Nrg1 were dramatically reduced by 35 minutes. In contrast, Nrg1 remained stable upwards of 65 minutes in a *pkc1Δ/pkc1Δ* mutant (Fig 6C), similar to the elevated levels of *NRG1* transcript that was observed in the Nanostring profile in response to serum (Fig 6B). Taken together, our results support a model in which Pkc1 promotes Cyr1 activity to modulate cAMP signaling and morphogenesis.

### Pkc1 and Ras1 co-regulate cAMP signaling, and Pkc1 acts through multiple pathways to enable filamentation

**Fig 7:**
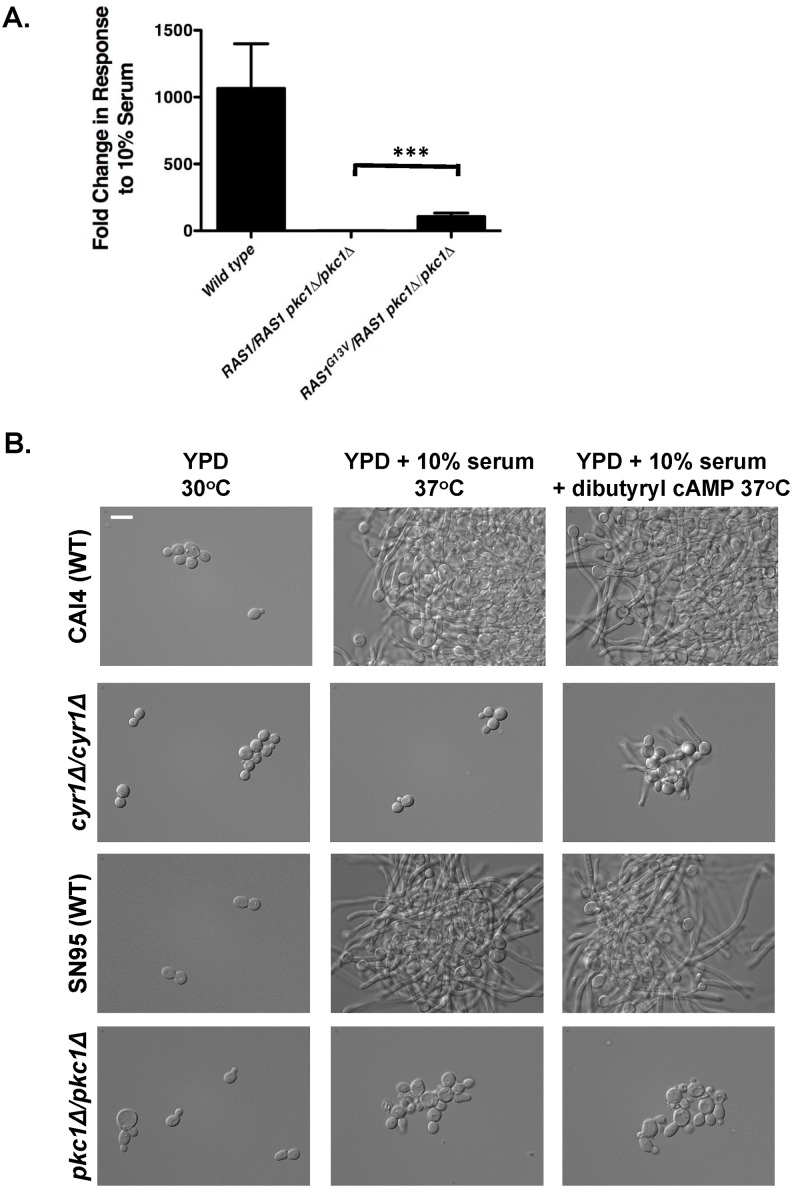
Pkc1 and Ras1 converge on regulating cAMP signaling, and Pkc1 governs morphogenesis through additional effectors. **(A)** Hyperactivation of Ras1 partially restores *HWP1* transcript levels in a mutant lacking Pkc1. Strains were subcultured to log phase in YPD at 30°C or YPD + 10% serum at 37°C. The transcript levels of *HWP1* and *IHD1* in YPD + 10% serum at 37°C were first normalized to *GPD1* and then normalized to the level in YPD at 30°C. Data are plotted as means ± SD for triplicate samples and are representative of two independent experiments. ***, *p* < 0.005 (Student *t* test). **(B)** Activation of cAMP signaling does not rescue filamentation in a mutant lacking Pkc1. Strains were grown in YPD at 30°C, YPD + 10% serum with or without 10 mg/mL of dibutyryl cAMP at 30°C for 3.5 hrs. Cells were imaged by DIC microscopy. The scale bar indicates 10 μm.

**Fig 8:**
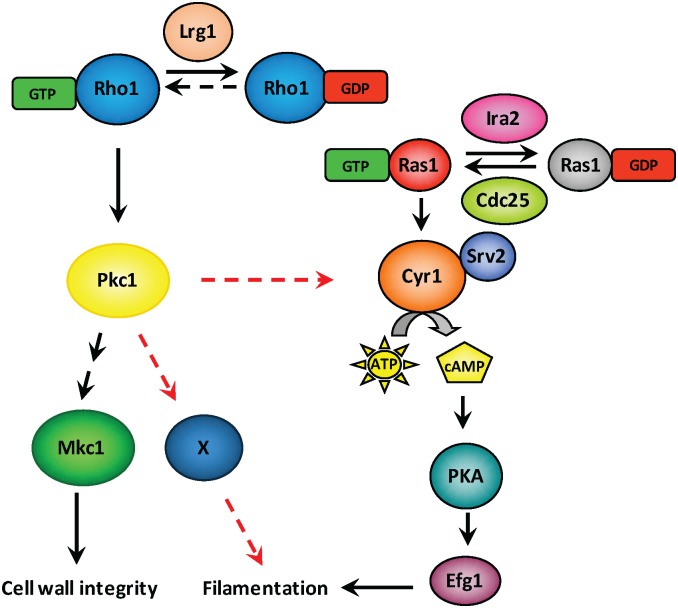
A schematic diagram depicting the regulation of filamentation by Rho1-Pkc1 signaling and cAMP-PKA signaling pathways. The Ras1-cAMP-PKA signaling cascade is a known master regulator of *C*. *albicans* filamentous growth. The Rho1-Pkc1 cell wall integrity pathway is previously reported as a key modulator of cell wall integrity through activation of a MAPK cascade that terminates with Mkc1. Key proteins involved in both signaling cascades are depicted along with black arrows showing connections between these regulators. Our work identifies a novel role for Pkc1 in governing *C*. *albicans* morphogenesis by directly or indirectly regulating Cyr1 function (red dashed arrow) and through distinct effector(s) remain to be identified (red dashed arrow).

Finally, we explored the functional relationship between Pkc1 and Ras1, and tested whether activation of cAMP-PKA signaling was sufficient to rescue filamentation in a strain lacking Pkc1. Quantification of the filamentous growth program by monitoring *HWP1* transcript levels revealed that introduction of the hyperactive *RAS1*^*G13V*^ allele in the *pkc1Δ/pkc1Δ* mutant partially restored the expression of *HWP1* in the presence of serum as compared to a *pkc1Δ/pkc1Δ* mutant alone (Fig 7A, Student’s t-test, *p* = 0.0022). These results suggest that Cyr1 is co-regulated by both Ras1 and Pkc1, since partial induction of the hyphal growth program still occurs in a *pkc1Δ/pkc1Δ* mutant. To determine whether filamentation driven by Pkc1 activity occurs solely through cAMP-PKA signaling, we tested whether the addition of dibutyryl cAMP, a non-hydrolysable cAMP analog, could rescue filamentous growth in a homozygous *PKC1* mutant. Supplementation of dibutyryl cAMP enabled filamentation in response to serum in a *cyr1Δ/cyr1Δ* mutant but not a *pkc1Δ/pkc1Δ* mutant, suggesting that Pkc1 has additional targets independent of cAMP signaling through which it regulates polarized growth (Fig 7B and S5 Fig). Thus, we propose that Pkc1 acts as a global regulator of *C*. *albicans* morphogenesis through regulation of the cAMP-PKA signaling cascade at or above Cyr1, and through a cAMP-independent pathway (Fig 8).

## Discussion

Morphogenetic transitions underpin virulence of diverse fungal pathogens. Our work has revealed novel regulators that control *C*. *albicans* morphogenesis in response to diverse host-relevant cues. Leveraging a genetic screen, we identified the Rho1 GAP, Lrg1, as a repressor of filamentation in *C*. *albicans* (Fig 1). Locking Rho1 in the active GTP-bound form by introducing a Q67L substitution or by deleting *LRG1* led to constitutive filamentation in the absence of inducing cues (Fig 1). Rho1 controls filamentation through the protein kinase Pkc1 (Fig 2), but in a manner that is independent of the canonical MAP kinase cascade (Fig 4). We also present the first link between the central regulators Pkc1 and Ras1 in functioning together to coordinate a cellular response, as Pkc1 and Ras1 modulate Cyr1 effects on morphogenesis in *C*. *albicans* (Figs 5–7). Thus, our results illuminate a new role for signaling through Lrg1, Rho1, and Pkc1 in orchestrating morphogenetic programs in response to diverse cues, and establish a novel link between Pkc1 and Ras1 in driving cellular morphogenesis (Fig 8).

Fungi utilize complex circuitry to control cellular morphology. For *C*. *albicans*, the number of positive regulators of morphogenesis that enable filamentation in response to cues vastly exceeds the number of negative regulators that maintain the yeast form of growth in the absence of inducing cues [16]. Lrg1 is distinct among the repressors of filamentation that have been identified to date. Only a handful of repressors have being well characterized, many of which are DNA binding proteins such as Rfg1 [39], Sfl1 [40], Nrg1 [41], and Tup1 [24]. Other than transcriptional repressors, cell cycle proteins feature as the most prominent repressors of filamentation [16]. In contrast, Lrg1 is a Rho1 GAP, which negatively regulates Rho1 function by stimulating its GTPase activity, thereby converting Rho1 from an active, GTP-bound state to an inactive, GDP-bound state. Our results suggest that Lrg1 represses filamentous growth by downregulating Rho1 activity. In *S*. *cerevisiae*, Lrg1 has been implicated as a negative regulator of haploid invasive growth [28], highlighting conserved cellular circuitry controlling filamentous growth in distinct yeast species. The essentiality of Pkc1 and Cyr1 in *S*. *cerevisiae* may have obscured possible roles of these regulators in morphogenesis. The extensive rewiring of morphogenetic circuitry that has been observed between *C*. *albicans* and *S*. *cerevisiae* [16], suggests that altered functional relationships among these regulators are likely to be uncovered.

Pkc1 is a central hub in cellular circuitry and is fundamental to coordinating sensing of environmental cues and orchestrating cellular responses via the cell wall integrity pathway. Our discovery that Pkc1 is required for *C*. *albicans* filamentation in response to diverse cues, but that the MAP kinase cascade is dispensable, highlights a novel role for Pkc1 as a global regulator of filamentous growth while implicating alternative downstream effectors. In *S*. *cerevisiae*, Pkc1 controls multiple cellular processes in addition to the downstream MAP kinase cascade, including activation of DNA integrity checkpoints [42], microtubule functions [43], glycerol metabolism [44, 45], as well as organization of the actin cytoskeleton [46]. In *C*. *albicans*, the impact of these processes on filamentation may suggest clues as to mechanisms through which Pkc1 affects morphogenesis independent of cAMP-PKA signaling. In *C*. *albican*s, inhibition of DNA replication with hydroxyurea or disruption of microtubules with nocodazole triggers polarized growth [47], and thus alterations in these processes are unlikely to account for the filamentation defect upon loss of Pkc1 kinase activity. In contrast, destabilization of actin cables blocks filamentous growth, suggesting that Pkc1 may enable filamentation by controlling remodeling of the actin cytoskeleton [48]. Previous studies have established a link between actin and filamentous growth as Cyr1 co-purifies with the cyclase-associated protein Srv2 as well as with monomeric actin (G-actin), which together increase cAMP synthesis in response to hyphal signals [49]. Thus, it would be interesting to further elucidate the role of actin in filamentation governed through Pkc1 signaling.

The central importance of morphogenesis for fungal nutrient foraging, virulence, and immune evasion, may underpin the elaboration of complex morphogenetic circuitry. It is striking that despite many pathways that operate in parallel, there is little functional redundancy at levels above transcriptional regulation. For example, the classic central pathway that governs *C*. *albican*s morphogenesis is the cAMP-PKA cascade [10, 36]. Deletion of genes encoding positive regulators of the pathway, such as Ras1 or the adenylyl cyclase, blocks filamentation in response to diverse cues. We discovered that the Lrg1-Rho1-Pkc1 pathway is also required for filamentation in response to diverse cues, and seems to operate in part through the co-regulation of cAMP production. Although it has been well established that PKA signals through the transcription factor Efg1 to mediate the morphological transition, alternate downstream targets of Pkc1 remain elusive (Fig 8). Notably, Pkc1 has been shown to play an active role in actin nucleation [50], and it phosphorylates Bni1 in *S*. *cerevisiae* [51]. Bni1 and Bnr1 are the two formins that participate in the nucleation of actin [52]. Genetic compromise of both formins in *C*. *albicans* has been shown to block filamentation in response to serum [53], providing a possible functional connection between Pkc1 and actin dynamics.

Pkc1 regulates diverse fungal cellular processes and is emerging as a promising drug target for the development of antifungal therapy. In addition to morphogenesis, Pkc1 plays a key role in mediating drug resistance in *C*. *albicans* [54]. In fact, a number of Pkc1 inhibitors were identified in a screen for small molecules that abrogate azole resistance of a *C*. *albicans* clinical isolate [54], suggesting that targeting Pkc1 holds great therapeutic potential in combination with existing antifungal drugs. Kinases provide tractable targets for pharmacological inhibition and a number of PKC inhibitors have advanced in clinical trials for a number of diseases, including oncology, congestive heart failure, bipolar disorder, and diabetic retinopathy [55]. However, targeting mammalian PKC is complicated by the existence of multiple isoforms, encoded by seven closely related genes [55]. Fungal Pkc1 is biochemically and functionally distinct from the mammalian PKCs, yet the catalytic domain is highly conserved between yeast and human [32]. However, it is possible to specifically target Pkc1 in fungi, as demonstrated by the discovery of cercosporamide, a natural product identified in a screen for selective inhibitors against *C*. *albicans* Pkc1 [56]. Therefore, Pkc1 is a major virulence factor in *C*. *albicans* with a significant role in the regulation of morphogenesis and drug resistance, and represents a feasible target for the development of novel antifungal drugs. In the broader perspective, characterizing virulence factors opens new target space for antifungal drug development, and new opportunities for chemical diversity in therapeutics as the cognate inhibitors are not typically explored by conventional screening approaches.

## Materials and Methods

### Strains and Reagents

All *C*. *albicans* strains were archived in 25% glycerol and stored at -80°C. Overnight cultures were grown in YPD (1% yeast extract, 2% bactopeptone, 2% glucose) at 30°C. 2% agar was added for solid media. Strains were constructed according to standard protocols. Strain construction is described in S1 Text and strains used in this study are listed in S2 Table. Doxycycline (DOX, BD Biosciences #631311) was formulated at 20 mg/ml in water and used at a final concentration of 0.05 μg/ml as specified. 1-Naphthyl-PP1 (1-NA-PP1, Cayman Chemical #221243-82-9) was formulated at 5 mM in DMSO (Sigma-Aldrich) and used at a final concentration of 5 μM. Geldanamycin (GdA, Cedarlane, ant-gl-5) was formulated at 5 mM in DMSO and used at a final concentration of 10 μM. N6,2’-O-Dibutyryladenosine 3’, 5’-cyclic monophosphate sodium salt (dibutyryl cAMP, Sigma-Aldrich, D0627) was formulated at 100 mg/ml and used at a final concentration of 10 mg/ml.

### Culturing Conditions

To deplete *RHO1*, strains were grown overnight at 30°C in YPD. Stationary phase cultures were split, adjusted to an OD_600_ of 0.1 where one culture was treated with 0.05 μg/ml of doxycycline, while the other was left untreated. Cells were grown for 16 hrs and were split again. The cells were adjusted to an OD_600_ of 0.2 and grown for 6 hrs. To assess mutant phenotypes in response to filament-inducing cues by microscopy, cells were subcultured for 4 hrs at 37°C in YPD only, YPD with 10% v/v heat-inactivated newborn calf serum (NBCS, Gibco #26010–066), RPMI medium (Gibco #11875–093), Spider medium (1% mannitol, 1% nutrient broth and 0.2% K_2_HPO_4_), or 4 hrs at 30°C in YPD with 10 μM geldanamycin. To monitor expression of hyphal genes in response to filament-inducing cues by qRT-PCR, cells were subcultured in YPD at 30°C or YPD with 10% NBCS at an OD_600_ of 0.2 and grown for 3.5 hrs.

### Plasmid Construction

Cloning procedures were performed following standard protocols. Plasmid construction is described in S1 Text and plasmids used in this study are listed in S3 Table. The absence of nonsynonymous mutations on the plasmid was verified by sequencing. Primers used in this study are listed in S4 Table.

### Arrayed Morphology Screen

A *C*. *albicans* homozygous transposon insertion mutant library [22, 23, 57–60] was screened by microscopy. Overnight cultures were set up by transferring colonies from solid medium with a multichannel pipette and grown statically in 200 μl YPD in 96-well plates at 30°C. Approximately 0.5 μl of the cells were then transferred via 96-well pinner into rich medium (YPD) and incubated at 30°C for 8 hrs under static conditions. Images of potential hits were captured on a Zeiss Axio Observer.Z1 (Carl Zeiss) using 10x and 40x magnifications. The mutants were scored for degree of filamentation on a scale from 0 (yeast), 1 (chained yeast), 2 (short filaments) or 3 (long filaments). To validate the phenotypes, overnight cultures were set up for the strains of interest in YPD at 30°C and grown shaking at 200 rpm. Images were captured using differential interference contrast (DIC) microscopy on a Zeiss Imager M1 upright microscope and AxioCam Mrm with AxioVision 4.7 software at 100x magnification.

### Quantitative Reverse Transcription PCR (qRT-PCR)

To prepare samples for RNA extraction, 10 ml of subcultures were harvested by centrifugation at 3000 rpm for 5 min. The pellet was flash-frozen and stored at -80°C overnight. RNA was isolated using the QIAGEN RNeasy kit and cDNA was generated using the AffinityScript cDNA synthesis kit (Stratagene). qRT-PCR was carried out using the Fast SYBR Green Master Mix (Thermo Fisher Scientific) in 384-well plate with the following cycle conditions: 95°C for 10 min, repeat 95°C for 10 sec, 60°C for 30 sec for 40 cycles. The melt curve was completed with the following cycle conditions: 95°C for 10 sec and 65°C for 5 seconds with an increase of 0.5°C per cycle up to 95°C. All reactions were done in triplicate. Data were analyzed in the Bio-Rad CFX manager 3.1.

### Active Ras1 Pull-down Assay

Active GTP-bound Ras1 was isolated using the Active Ras Pull-Down and Detection Kit (Pierce) following protocol as described previously [11].

### Nanostring

Nanostring nCounter (Nanostring Technologies) analysis was used to quantify *C*. *albicans* gene expression. After 3.5 h cells were harvested and fungal RNA was isolated using MasterPure Yeast RNA Purification Kit (Epicentre). Each Nanostring reaction mixture contained 70 ng fungal RNA, hybridization buffer, reporter and capture probes. Overnight hybridization of RNA with probes at 65°C preceded sample preparation using Nanostring prep station. Targets were counted on the nCounter using 255 fields of view per sample [61]. Raw counts for Cyr1 regulated transcripts (*HWP1*, *ECE1*, *HGC1*, *HYR1*, *RBT1*, *TDH3*, *NRG1*, *ACO1*, *ALD5*, *ALS4*, and *YWP1*) were normalized using the functions “calcNormFactors” (‘method’ set to “TMM”), “estimateCommonDisp” (‘verbose’ set to “TRUE”) and “estimateTagwiseDisp” (‘trend’ set to”none”) in the “edgeR” (v.3.14.0) package in R (v.3.3.0) (R Foundation for Statistical Computing, Vienna, Austria). The arithmetic mean was taken from two biological replicates. Heat maps were created using Z-scoring of normalized Nanostring counts of selected yeast- and hyphal-specific genes using the “heatmap.2” (‘scale’ set to “row”) function in the “gplots” package (v.3.0.1) in R.

### Minimum Inhibitory Concentration Assay

Antifungal susceptibility testing was performed in flat bottom, 96-well microtitre plates (Sarstedt) using a standard broth microdilution protocol [62]. Minimum inhibitory concentration (MIC) assays were set up in 2-fold serial dilutions of calcofluor white in a final volume of 200 μl per well. The calcofluor white gradient was from 125 μg/ml down to 0.5 μg/ml. Where applicable, DMSO is added to a final concentration of 0.05%, and 1-NA-PP1 is added to a final concentration of 5 μM. Cell densities of overnight cultures were determined by measuring 100 μl of the culture in the 96-well plate in duplicates and dilutions were prepared such that ~10^3^ cells were inoculated into each well. Plates were incubated in the dark at 30°C for 48 hrs, at which point the plates were sealed with tape and agitated to suspend the re-suspend the cells. Optical density was determined at 600 nm using a spectrophotometer (Molecular Devices) and was corrected for background from the corresponding medium. Each strain was tested in duplicate in three biological replicates. Growth was quantitatively displayed with color using the program Java TreeView 1.1.3 (http://jtreeview.sourceforge.net).

## Supporting Information

S1 Table Negative regulators of filamentation for the arrayed morphology screens. Mutants were scored for degree of filamentation on a scale from 0 (yeast), 1 (chained yeast), 2 (short filaments), or 3 (long filaments).
(XLSX)

S2 Table Strains used in this study. (DOCX)

S3 Table Plasmids used in this study. (DOCX)

S4 Table Primers used in this study. (XLSX)

S1 Text Supplemental Methods and Supplemental References. (DOCX)

S1 Fig Transcriptional repression of *RHO1* in *tetO-RHO1/rho1Δ* is achieved with 0.05 μg/ml doxycycline. Overnights were subcultured for 24 hrs in the presence or absence of doxycycline. The strains were subcultured again in the same conditions for 4 hrs. cDNA was prepared from total RNA for qRT-PCR. The transcript level of *RHO1* was monitored and normalized to *GPD1*. Data are plotted as means ± SD for triplicate samples and are representative of two independent experiments.
(TIFF)

S2 Fig The osmotic stabilizer sorbitol does not restore filamentation of a mutant lacking Pkc1 in response to serum. Strains were grown in YPD + 1M sorbitol at 30°C with or without 10% serum for 3.5 hrs. Cells were imaged by DIC microscopy. The scale bar indicates 10 μm.
(TIFF)

S3 Fig The *PKC1* gatekeeper mutant is sensitive to ATP analogue 1-NA-PP1 and high temperature. Calcofluor white minimum inhibitory concentration (MIC) assays were conducted in YPD medium in the presence of 0.05% DMSO or in the presence of 5 μM 1-NA-PP1 as indicated. Growth was measured by absorbance at 600 nm after 48 hours incubation at 30°C or 37°C as specified. Optical densities were averaged for duplicate measurements. Data was quantitatively displayed with colour using Treeview (see colour bar).
(TIFF)

S4 Fig Cyr1 signaling is blocked in the absence of Pkc1. Strains were grown in YPD+10% serum at 37°C for 3.5 hrs at 200 rpm. Total RNA was analyzed on the Nanostring nCounter system. Heat maps were created using Z-scores of normalized Nanostring counts comparing strains in YPD+10% serum at 37°C.
(TIFF)

S5 Fig Increase in cAMP signaling rescues filamentation in a mutant lacking Cyr1. Strains were grown in YPD at 30°C, YPD + 10% serum with or without 10 mg/ml of dibutyryl cAMP at 30°C for 6 hrs.
(TIFF)
