# Supplementary material for: Signaling through Lrg1, Rho1 and Pkc1 Governs Candida albicans Morphogenesis in Response to Diverse Cues
Source: PLoS Genet. 2016 Oct 27;12(10):e1006405. doi: 10.1371/journal.pgen.1006405 (PMC5082861; doi:10.1371/journal.pgen.1006405)
Supplement: S2 Table — (DOCX) [file pgen.1006405.s002.docx]

**Table S2. *Candida albicans* strains used in this study.**

| Strain Name | Genotype or phenotype | Source |
| --- | --- | --- |
| CaLC1900 (DAY286) | *ura3::imm434/ura3::imm434 iro1/iro1::imm434 his1::hisG/his1::hisG arg4/arg4* | [1] |
| CaLC1891 | *ura3::imm434/ura3::imm434 iro1/iro1::imm434 his1::hisG/his1::hisG arg4/arg4*  *tup1∷Tn7-UAU1/tup1∷Tn7-URA3* | [2] |
| CaLC1897 | *ura3::imm434/ura3::imm434 iro1/iro1::imm434 his1::hisG/his1::hisG arg4/arg4*  *sfl1∷Tn7-UAU1/sfl1∷Tn7-URA3* | [2] |
| CaLC1901 | *ura3::imm434/ura3::imm434 iro1/iro1::imm434 his1::hisG/his1::hisG arg4/arg4*  *lrg1∷Tn7-UAU1/lrg1∷Tn7-URA3* | [2] |
| CaLC239 (SN95) | *arg4∆/arg4∆ his1∆/his1∆ URA3/ura3∆::imm434 IRO1/iro1::imm434* | [3] |
| CaLC206 | SN95 *CaTAR-FRT∷his1∆/his1∆* | [3] |
| CaLC3935 | CaLC206 *CaTAR-FRT-tetO-RHO1/rho1∷FRT* | This study |
| CaLC4501 | CaLC206 *CaTAR-FRT-tetO-RHO1/RHO1Q67L* | This study |
| CaLC948 | CaLC206 *PKC1/pkc1∷FRT* | [4] |
| CaLC1255 | CaLC206 *pkc1∷FRT/pkc1∷FRT* | [4] |
| CaLC1256 | CaLC206 *PKC1-FRT/pkc1∷FRT* | [4] |
| CaLC3076 | CaLC206 *PKC1M850G-FRT/pkc1∷FRT* | This study |
| CaLC4090 | SN95 *lrg1::FRT/lrg1::FRT* | This study |
| CaLC4375 | SN95 *lrg1::FRT/lrg1::FRT PKC1/pkc1∷FRT* | This study |
| CaLC896 | SN95 *bck1∷FRT/bck1∷FRT* | [4] |
| CaLC700 | SN95 *mck1∷FRT/mkc1∷FRT* | [4] |
| CaLC3869 | SN95 *NAT-GFP-RAS1G13V/RAS1* | This study |
| CaLC3844 | CaLC1255 *NAT-GFP-RAS1G13V/RAS1* | This study |
| CaLC75 (CAI4) | *ura3::imm434/ura3::imm434* | [5] |
| CaLC555 | CAI4 *cyr1::hisG-URA3-hisG/cyr1::hisG* | [6] |
| CaLC4132 | SN95 *NRG1-HA/NRG1* | This study |
| CaLC4185 | SN95 *pkc1::FRT/pkc1::FRT NRG1-HA/NRG1* | This study |
