# Supplementary material for: Signaling through Lrg1, Rho1 and Pkc1 Governs Candida albicans Morphogenesis in Response to Diverse Cues
Source: PLoS Genet. 2016 Oct 27;12(10):e1006405. doi: 10.1371/journal.pgen.1006405 (PMC5082861; doi:10.1371/journal.pgen.1006405)
Supplement: S3 Table — (DOCX) [file pgen.1006405.s003.docx]

**Table S3. Bacterial plasmids used in this study.**

| Strain Name | Description | Source |
| --- | --- | --- |
| pLC49 | *FLP-CaNAT, ampR* | [7] |
| pLC470 | pLC49 *CaPKC1*-KO, *ampR*, *NAT* | [4] |
| pLC605 | *CaTAr-FLP-CaNAT, ampR* | [8] |
| pLC379 | *GFP-GGG-V13Ras1*, *ampR*, *NAT* | [9] |
| pLC575 | *pFA-HA-HIS1, ampR* | [10] |
| pLC706 | pLC49 *CaPKC1, ampR, NAT* | This study |
| pLC770 | pLC49 *CaPKC1M850G, ampR, NAT* | This study |
| pLC765 | pLC49 *CaRHO1*, *ampR*, *NAT* | This study |
| pLC771 | pLC49 *CaRHO1T23N*, *ampR*, *NAT* | This study |
| pLC772 | pLC49 *CaRHO1Q67L*, *ampR*, *NAT* | This study |
