# Supplementary material for: Signaling through Lrg1, Rho1 and Pkc1 Governs Candida albicans Morphogenesis in Response to Diverse Cues
Source: PLoS Genet. 2016 Oct 27;12(10):e1006405. doi: 10.1371/journal.pgen.1006405 (PMC5082861; doi:10.1371/journal.pgen.1006405)
Supplement: S1 Text — (DOCX) [file pgen.1006405.s005.docx]

**Strain Construction**

**CaLC3935**: To generate a *RHO1* heterozygous deletion mutant in a strain carrying the transactivator, the NAT flipper cassette (pLC49) [7] was PCR amplified using primers oLC2836 and oLC2837 (4366 bp) and transformed into CaLC206. NAT resistant transformants were PCR tested with oLC2840 + oLC275 (654 bp) for upstream integration and oLC274 + oLC2843 (515 bp) for downstream integration. The *SAP2* promoter was induced to drive expression of FLP recombinase to excise the NAT flipper cassette. This strain was archived as CaLC2872. To regulate the expression of *RHO1*, the tetracycline-repressible transactivator, the tetO promoter, and the NAT flipper cassette were PCR amplified from pLC605 [8] using primers oLC2838 and oLC2860 (4957 bp) and transformed into CaLC2872. NAT resistant transformants were PCR tested with oLC2862 + oLC534 (522 bp) for upstream integration and oLC274 + oLC2842 (1223 bp) for downstream integration. The absence of WT *RHO1* promoter was verified with oLC2862 + oLC2842 (847 bp for WT promoter) and the presence of a deleted *RHO1* allele was verified with oLC2862 + oLC2843 (760 bp). The *SAP2* promoter was induced to drive expression of FLP recombinase to excise the NAT flipper cassette.

**CaLC4501**: To introduce the Q67L mutation into *RHO1*, the *RHO1* complementation vector carrying the Q67L mutation was released from pLC772 with BssHII and transformed into CaLC3935. NAT resistant transformants were PCR tested with oLC275 + oLC2841 (1076 bp) for upstream integration and oLC274 + oLC2868 (483 bp) for downstream integration. The presence of WT *RHO1* promoter was verified using primers oLC2864 + oLC2843 (1159 bp for WT promoter; 562 bp for the absence of WT promoter) and the presence of a *tetO-RHO1* allele was verified with oLC2862 + oLC534 (522 bp). The *SAP2* promoter was induced to drive expression of FLP recombinase to excise the NAT flipper cassette.

**CaLC3076**: To introduce the M850G mutation into *PKC1*, the *PKC1* complementation vector carrying the M850G mutation was released from pLC770 with BssHII and transformed into CaLC1255. NAT resistant transformants were PCR tested with oLC275 + oLC1042 (660 bp) for upstream integration and oLC274 + oLC1030 (555 bp) for downstream integration. The *SAP2* promoter was induced to drive expression of FLP recombinase to excise the NAT flipper cassette.

**CaLC4090**: To generate a *LRG1* heterozygous deletion mutant, the NAT flipper cassette (pLC49) [7] was PCR amplified using primers oLC3976 and oLC3977 (4366 bp) and transformed into CaLC239. NAT resistant transformants were PCR tested with oLC3978 + oLC275 (bp) for upstream integration and oLC274 + oLC3979 (bp) for downstream integration. The *SAP2* promoter was induced to drive expression of FLP recombinase to excise the NAT flipper cassette.To generate a *LRG1* homozygous deletion mutant, the NAT flipper cassette (pLC49) [7] was PCR amplified using primers oLC3976 and oLC3977 (4366 bp) and transformed into the *LRG1* heterozygous deletion mutant. NAT resistant transformants were PCR tested with oLC3972 + oLC275 (bp) for upstream integration and oLC274 + oLC3973 (bp) for downstream integration. The absence of WT *LRG1* allele was verified with oLC3980 + oLC3981 (bp), the absence of a band confirmed absence of a wild type allele. The *SAP2* promoter was induced to drive expression of FLP recombinase to excise the NAT flipper cassette.

**CaLC4375**: To generate a *PKC1* heterozygous deletion mutant in a strain lacking *LRG1*, the *PKC1* deletion construct was released from pLC470 [4] using BssHII and transformed into CaLC4090. NAT resistant transformants were PCR tested with oLC950 + oLC275 (850 bp) for upstream integration and oLC274 + oLC951 (730 bp) for downstream integration. The *SAP2* promoter was induced to drive expression of FLP recombinase to excise the NAT flipper cassette.

**CaLC3869**: To introduce the G13V mutation into *RAS1* and N-terminally tag Ras1 with GFP in SN95, the GFP-Ras1 construct carrying the G13V mutation was released from pLC379 [9] with Sac1 and Kpn1 and transformed into CaLC1255. NAT resistant transformants were PCR tested with oLC275 + oLC554 (465 bp) for upstream integration and oLC559 and oLC563 (1246 bp) for downstream integration. The integrated *RAS1* mutant allele was PCR amplified with oLC274 + oLC563 (~2 kb) and presence of the G13V mutation was confirmed by sequence analysis using oLC562.

**CaLC3844**: To introduce the G13V mutation into *RAS1* and N-terminally tag Ras1 with GFP in a strain lacking *PKC1*, the GFP-Ras1 construct carrying the G13V mutation was released from pLC379 [9] with Sac1 and Kpn1 and transformed into CaLC1255. NAT resistant transformants were PCR tested with oLC275 + oLC554 (465 bp) for upstream integration and oLC559 and oLC563 (1246 bp) for downstream integration. The integrated *RAS1* mutant allele was PCR amplified with oLC274 + oLC563 (~2 kb) and presence of the G13V mutation was sequenced with oLC562.

**CaLC4132**: To C-terminally HA tag Nrg1 in SN95 (WT), the *NRG1-HA-HIS* construct was amplified using pLC575 with oLC4007/oLC4008 (1724 bp) and transformed into CaLC239. Proper integration was verified by colony PCR using primers oLC3280/oLC3267 (2884 bp). Expression was subsequently confirmed by Western blot.

**CaLC4185**: To C-terminally HA tag Nrg1 in a mutant lacking Pkc1, the *NRG1-HA-HIS* construct was amplified using pLC575 with oLC4007/oLC4008 (1724 bp) and transformed into CaLC1255. Proper integration was verified by colony PCR using primers oLC3280/oLC3267 (2884 bp). Expression was subsequently confirmed by Western blot.

**Plasmid Construction**

**pLC706:** This is a construct to complement WT *CaPKC1* in *C. albicans*. Homology downstream of *PKC1* was amplified from SC5314 genomic DNA with primers oLC1029 and oLC1030 and cloned into pLC49 at NotI and SacII. The presence of the inserts was determined by PCR with oLC274 and oLC1030 (538 bp) as well as with oLC1029 and oLC244 (473 bp). Downstream homology was sequenced verified with oLC244 and oLC274. *PKC1* with a promoter region and some of the terminator region was amplified from SC5314 genomic DNA with oLC1027/oLC1028 (4138 bp) and cloned into pLC49 containing the downstream homology of *PKC1* at ApaI. The presence of the inserts were tested by PCR with oLC243 and oLC956 (942 bp) as well as with oLC275 and oLC1042 (605 bp). Upstream homology and ORF was sequence verified with oLC243, oLC1027, oLC1035, oLC1036, oLC1037, oLC1038, oLC1039, oLC1040, and oLC1041. Downstream homology was re-verified with oLC244. The reconstitution construct can be liberated by digestion with BsshII.

**pLC770**: This is a construct to complement mutant *CaPKC1* carrying the M850G mutation in *C. albicans*. This plasmid is based on pLC706 but harbors a mutation in Pkc1 (M850G) that will not affect the kinase activity but render the kinase susceptible to 1-NA-PP1. This mutation was introduced by site-directed mutagenesis with primers oLC2998 and oLC2999. The clone was sequence verified with the following primers: oLC243, oLC1027, oLC1035, oLC1036, oLC1037, oLC1038, oLC1039, oLC1040, and oLC1041. The reconstitution construct can be liberated by digestion with BsshII.

**pLC765**: This is a construct to complement WT *CaRHO1* in *C. albicans*. Homology downstream of *RHO1* was amplified from SC5314 genomic DNA with primers oLC2867 and oLC2868 (351 bp) and cloned into pLC49 at NotI and SacII. The presence of the inserts were tested by PCR with oLC274 and oLC244 (550 bp). Downstream homology was sequenced verified with oLC274. *RHO1* with a promoter region and some of the terminator region was amplified from SC5314 genomic DNA with oLC2862 and oLC2866 (1557 bp) and cloned into pLC49 containing the downstream homology of *RHO1* at ApaI. The presence of the inserts were tested by PCR with oLC243 and oLC2863 (296 bp) as well as with oLC275 and oLC2865 (356 bp). Upstream homology and ORF was sequence verified with oLC243 and oLC275. The reconstitution construct can be liberated by digestion with BsshII.

**pLC772**: This is a construct to complement mutant *CaRHO1* carrying the Q67L mutation in *C. albicans*. This is based on pLC765 but harbors a mutation in Rho1 (Q67L). This mutation was introduced by site-directed mutagenesis with primers oLC2996 and oLC2997. The clone was sequence verified with oLC2864. The reconstitution construct can be liberated by digestion with BsshII.

**Supplemental References:**

1. Davis DA, Bruno VM, Loza L, Filler SG, Mitchell AP (2002) Candida albicans Mds3p, a conserved regulator of pH responses and virulence identified through insertional mutagenesis. Genetics 162: 1573-1581.

2. Enloe B, Diamond A, Mitchell AP (2000) A single-transformation gene function test in diploid Candida albicans. J Bacteriol 182: 5730-5736.

3. Noble SM, Johnson AD (2005) Strains and strategies for large-scale gene deletion studies of the diploid human fungal pathogen Candida albicans. Eukaryot Cell 4: 298-309.

4. LaFayette SL, Collins C, Zaas AK, Schell WA, Betancourt-Quiroz M, et al. (2010) PKC signaling regulates drug resistance of the fungal pathogen Candida albicans via circuitry comprised of Mkc1, calcineurin, and Hsp90. PLoS Pathog 6: e1001069.

5. Fonzi WA, Irwin MY (1993) Isogenic strain construction and gene mapping in Candida albicans. Genetics 134: 717-728.

6. Jain P, Akula I, Edlind T (2003) Cyclic AMP signaling pathway modulates susceptibility of candida species and Saccharomyces cerevisiae to antifungal azoles and other sterol biosynthesis inhibitors. Antimicrob Agents Chemother 47: 3195-3201.

7. Morschhauser J, Michel S, Staib P (1999) Sequential gene disruption in Candida albicans by FLP-mediated site-specific recombination. Mol Microbiol 32: 547-556.

8. Leach MD, Cowen LE (2014) Membrane fluidity and temperature sensing are coupled via circuitry comprised of Ole1, Rsp5, and Hsf1 in Candida albicans. Eukaryot Cell 13: 1077-1084.

9. Shapiro RS, Uppuluri P, Zaas AK, Collins C, Senn H, et al. (2009) Hsp90 orchestrates temperature-dependent Candida albicans morphogenesis via Ras1-PKA signaling. Curr Biol 19: 621-629.

10. Lavoie H, Sellam A, Askew C, Nantel A, Whiteway M (2008) A toolbox for epitope-tagging and genome-wide location analysis in Candida albicans. BMC Genomics 9: 578.
