# Supplementary figures and images for: Signaling through Lrg1, Rho1 and Pkc1 Governs Candida albicans Morphogenesis in Response to Diverse Cues
Source: PLoS Genet. 2016 Oct 27;12(10):e1006405. doi: 10.1371/journal.pgen.1006405 (PMC5082861; doi:10.1371/journal.pgen.1006405)

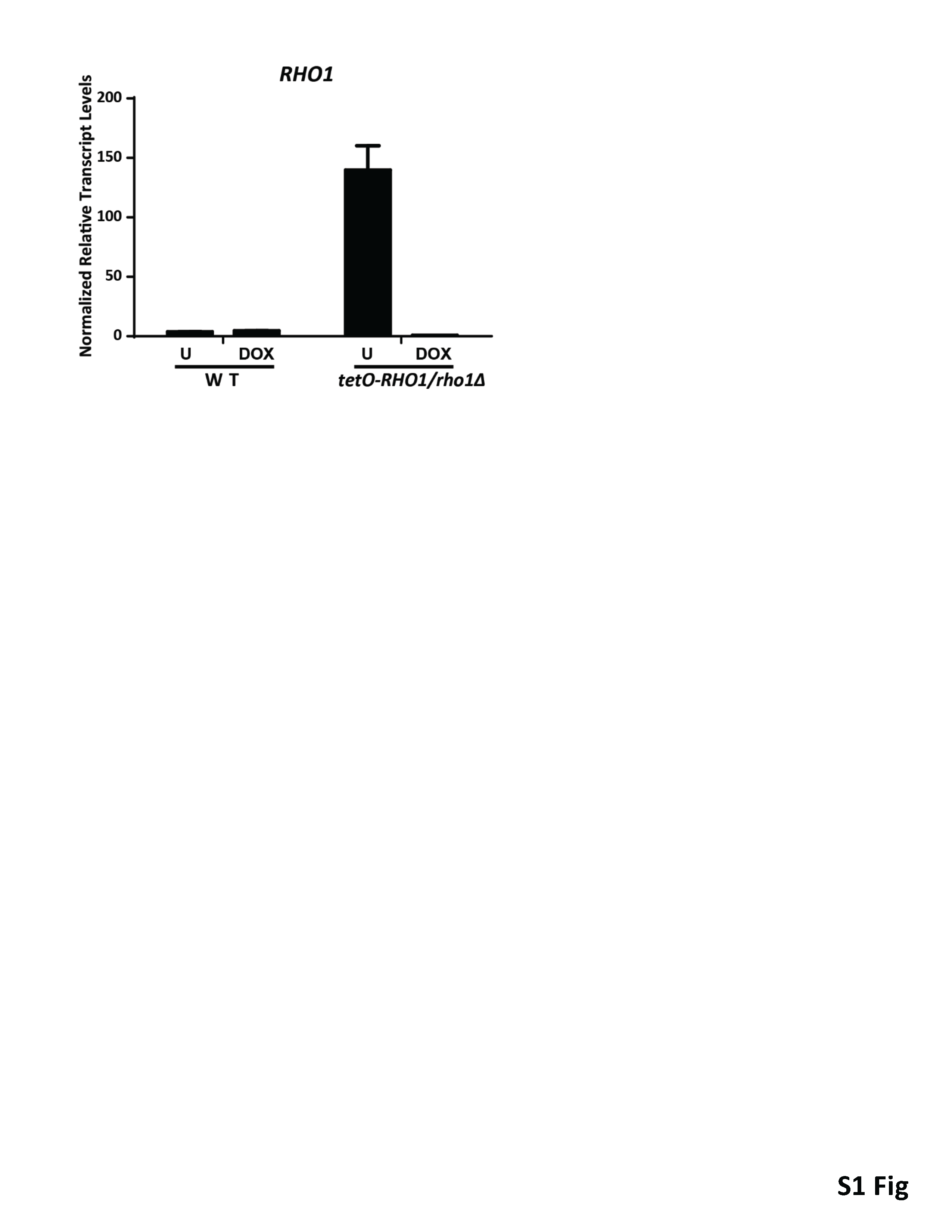

Supplement: S1 Fig — Overnights were subcultured for 24 hrs in the presence or absence of doxycycline. The strains were subcultured again in the same conditions for 4 hrs. cDNA was prepared from total RNA for qRT-PCR. The transcript level of RHO1 was monitored and normalized to GPD1. Data are plotted as means ± SD for triplicate samples and are representative of two independent experiments. (TIFF) [file pgen.1006405.s006.tiff]

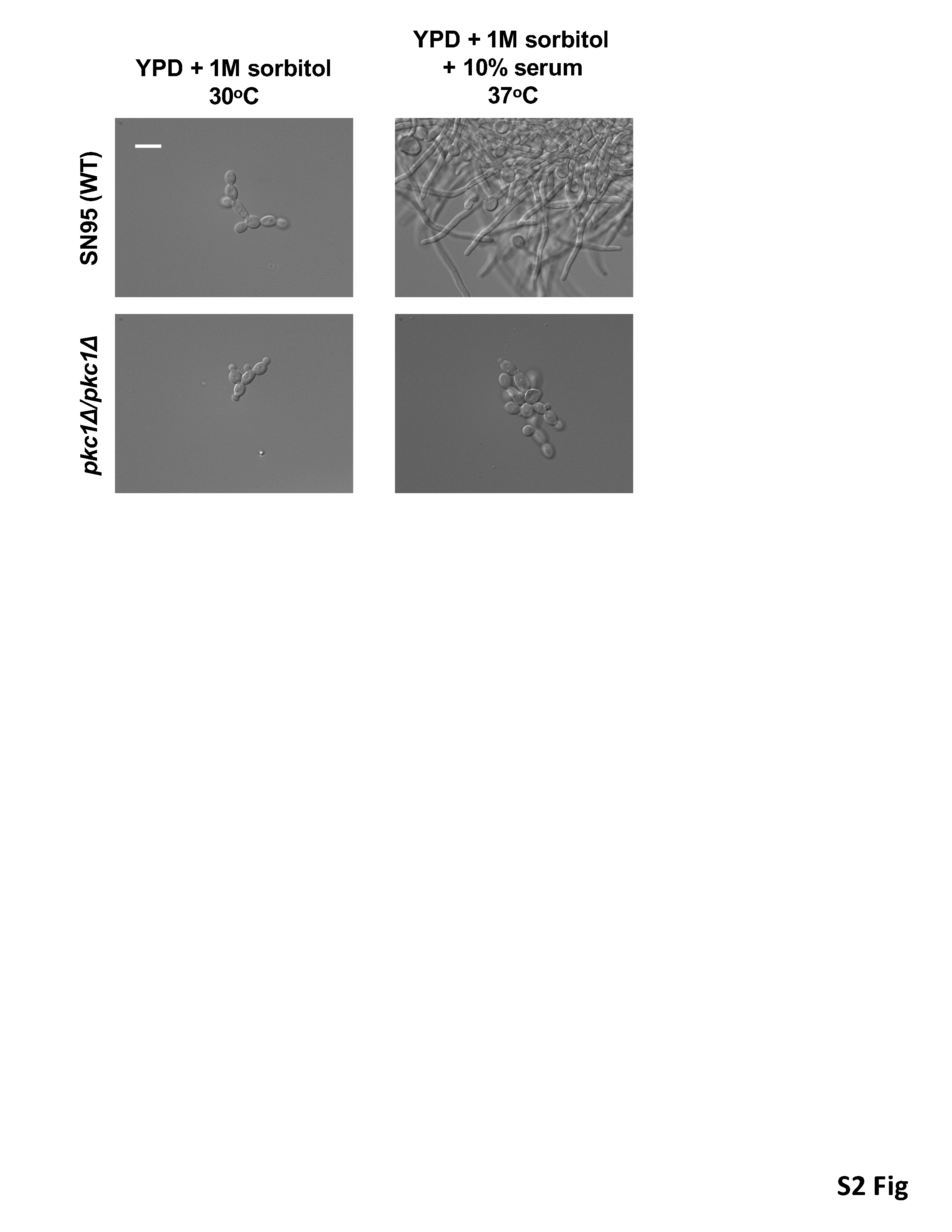

Supplement: S2 Fig — Strains were grown in YPD + 1M sorbitol at 30°C with or without 10% serum for 3.5 hrs. Cells were imaged by DIC microscopy. The scale bar indicates 10 μm. (TIFF) [file pgen.1006405.s007.tiff]

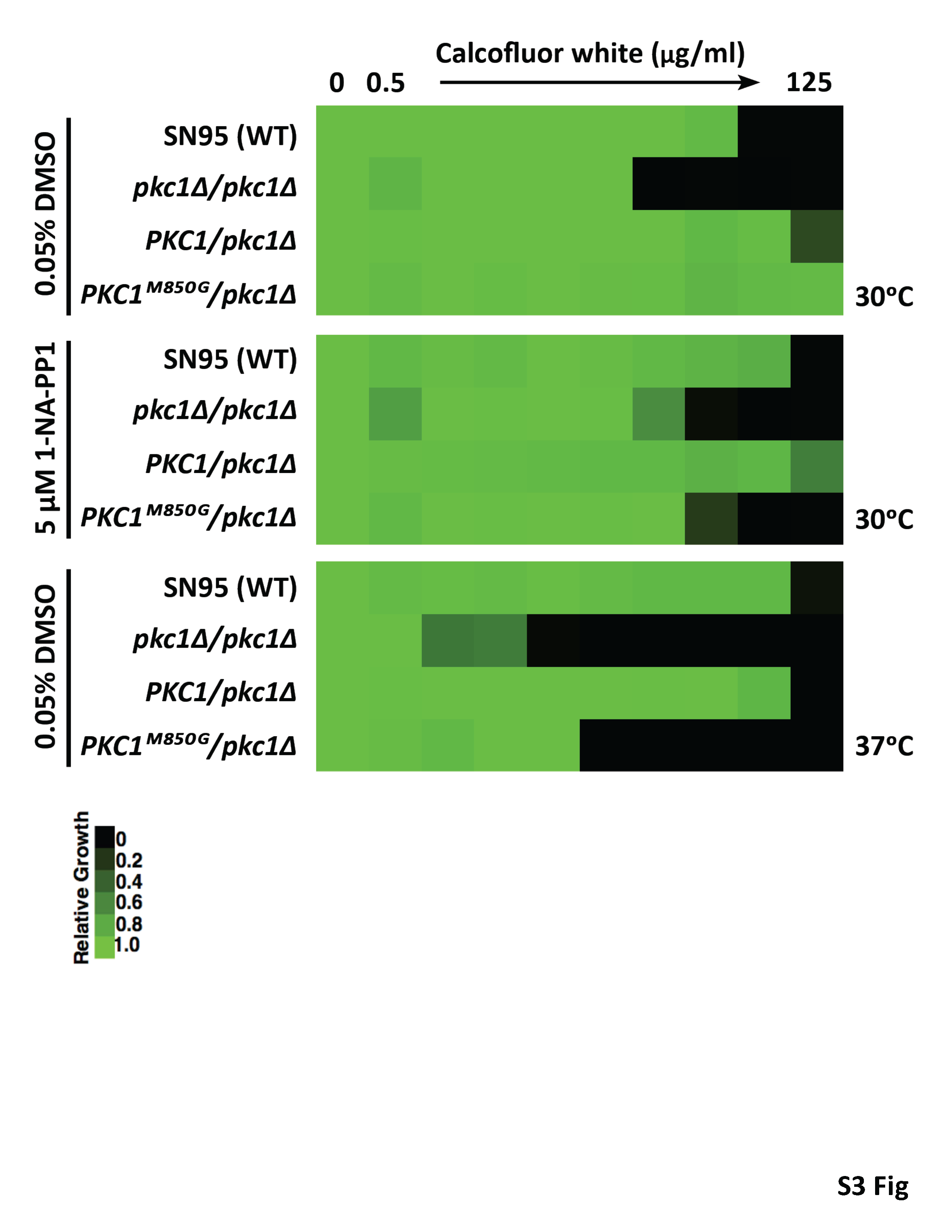

Supplement: S3 Fig — Calcofluor white minimum inhibitory concentration (MIC) assays were conducted in YPD medium in the presence of 0.05% DMSO or in the presence of 5 μM 1-NA-PP1 as indicated. Growth was measured by absorbance at 600 nm after 48 hours incubation at 30°C or 37°C as specified. Optical densities were averaged for duplicate measurements. Data was quantitatively displayed with colour using Treeview (see colour bar). (TIFF) [file pgen.1006405.s008.tiff]

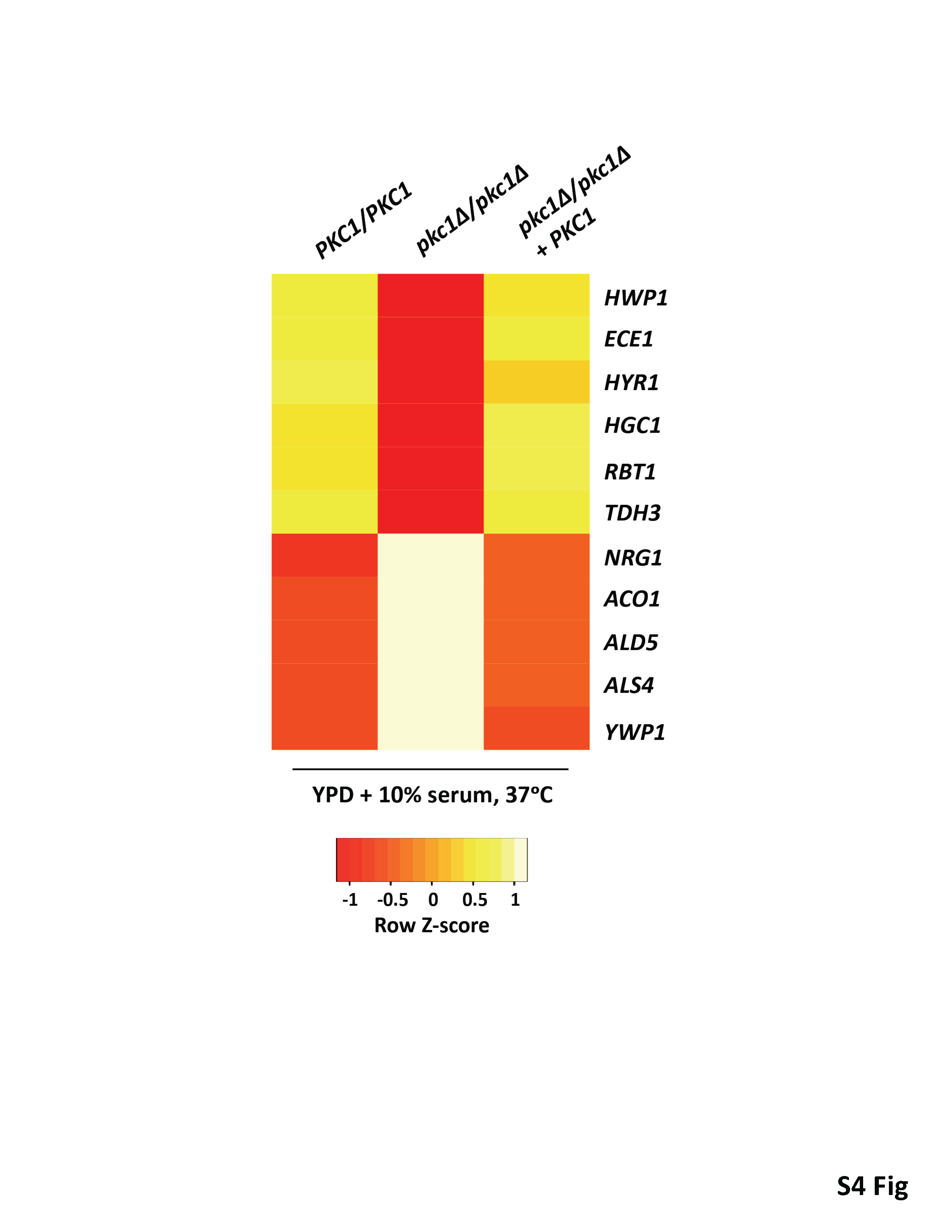

Supplement: S4 Fig — Strains were grown in YPD+10% serum at 37°C for 3.5 hrs at 200 rpm. Total RNA was analyzed on the Nanostring nCounter system. Heat maps were created using Z-scores of normalized Nanostring counts comparing strains in YPD+10% serum at 37°C. (TIFF) [file pgen.1006405.s009.tiff]

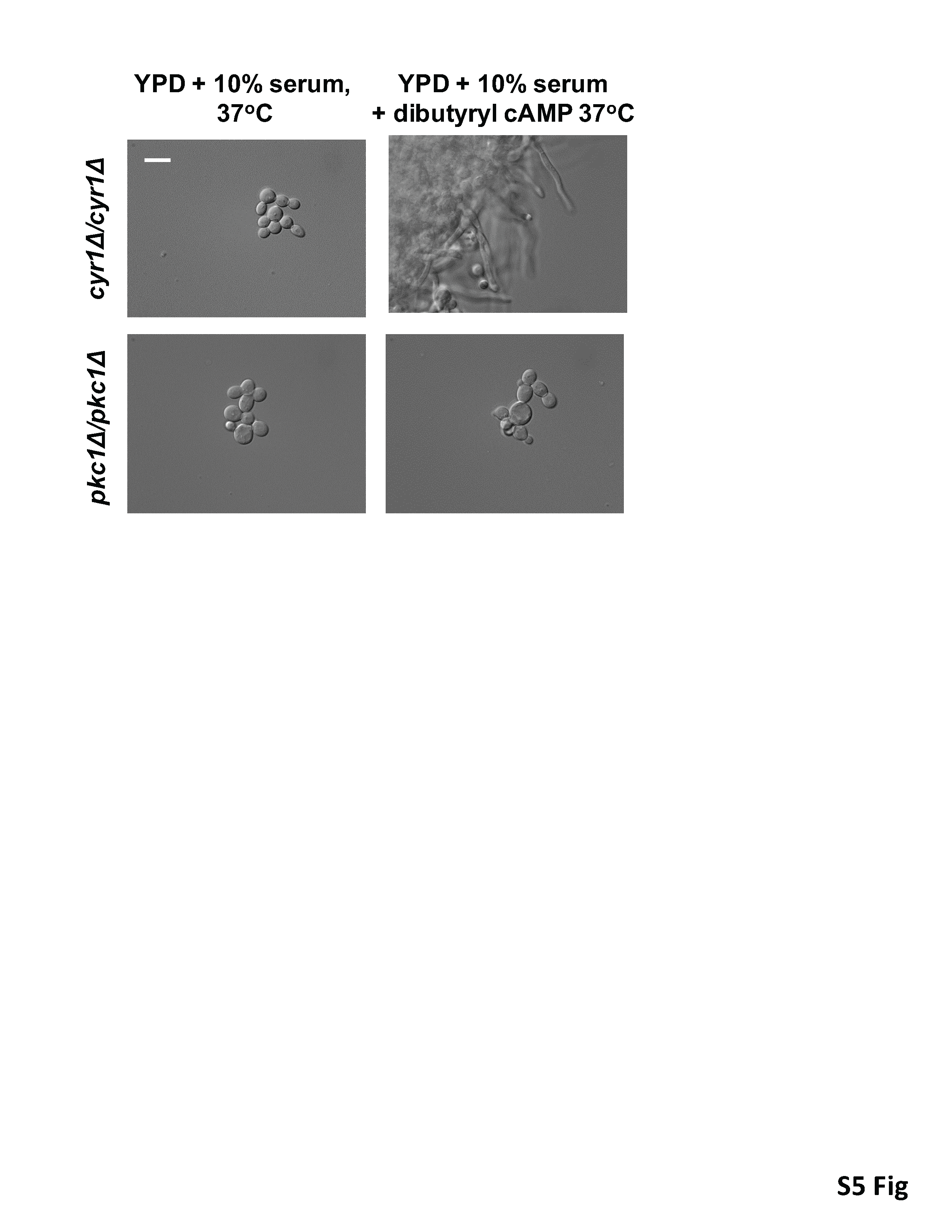

Supplement: S5 Fig — Strains were grown in YPD at 30°C, YPD + 10% serum with or without 10 mg/ml of dibutyryl cAMP at 30°C for 6 hrs. (TIFF) [file pgen.1006405.s010.tiff]
